# Biological Effects of Thermal Water-Associated Hydrogen Sulfide on Human Airways and Associated Immune Cells: Implications for Respiratory Diseases

**DOI:** 10.3389/fpubh.2019.00128

**Published:** 2019-06-05

**Authors:** Joana Viegas, Ana Filipa Esteves, Elsa M. Cardoso, Fernando A. Arosa, Marco Vitale, Luís Taborda-Barata

**Affiliations:** ^1^CICS-UBI–Health Sciences Research Centre, University of Beira Interior, Covilhã, Portugal; ^2^FCS–Faculty of Health Sciences, University of Beira Interior, Covilhã, Portugal; ^3^Escola Superior da Saúde, IPG–Instituto Politécnico da Guarda, Guarda, Portugal; ^4^DiMeC–Department of Medicine & Surgery, University of Parma, Parma, Italy; ^5^FoRST–Fondazione per la Ricerca Scientifica Termale, Rome, Italy; ^6^NuESA–Health & Environment Study Group, Faculty of Health Sciences, University of Beira Interior, Covilhã, Portugal; ^7^Department of Immunoallergology, CHUCB–Cova da Beira University Hospital Centre, Covilhã, Portugal

**Keywords:** sulfurous thermal waters, hydrogen sulfide, S-sulfhydration, allergic rhinitis, asthma, chronic obstructive pulmonary disease

## Abstract

Natural mineral (thermal) waters have been used for centuries as treatment for various diseases. However, the scientific background of such therapeutic action is mostly empiric and based on knowledge acquired over time. Among the various types of natural mineral waters, sulfurous thermal waters (STWs) are the most common type in the center of Portugal. STWs are characterized by high pH, poor mineralization, and the presence of several ions and salts, such as bicarbonate, sodium, fluoride, silica, and carbonate. Furthermore, these waters are indicated as a good option for the treatment of various illnesses, namely respiratory diseases (e.g., allergic rhinitis, asthma, and chronic obstructive pulmonary disease). From the sulfide species present in these waters, hydrogen sulfide (H_2_S) stands out due to its abundance. In healthy conditions, H_2_S-related enzymes (e.g., cystathionine β-synthase and cystathionine γ-lyase) are expressed in human lungs, where they have mucolytic, antioxidant, anti-inflammatory, and antibacterial roles, thus contributing to airway epithelium homeostasis. These roles occur mainly through S-sulfhydration, a post-translational modification through which H_2_S is able to change the activity of several targets, such as ion channels, second messengers, proteins, among others. However, in respiratory diseases the metabolism of H_2_S is altered, which seems to contribute somehow to the respiratory deterioration. Moreover, H_2_S has been regarded as a good biomarker of airway dysfunction and severity, and can be measured in serum, sputum, and exhaled air. Hence, in this review we will recapitulate the effects of STWs on lung epithelial-immune crosstalk through the action of its main component, H_2_S.

## Introduction

Natural mineral waters from thermal springs (thermal waters) are used in Europe since ancient Greece for hygiene and later for the treatment of several diseases (e.g., respiratory, skin, and musculoskeletal diseases). Nowadays these waters are also used beyond their conventional purposes, namely with preventive, anti-stress, and aesthetic functions. The classification of thermal waters (35–40°C) is based upon their physicochemical features, which allows their subdivision into sulfurous, salso-bromo-iodic, bicarbonate, and bicarbonate-sulfate waters (1, 2). In fact, a beneficial link between sulfurous thermal water (STWs) and clinical improvement of several illnesses has been suggested (3–6). Such beneficial effects may be due to analgesic, antioxidant (7), antibacterial (8), and anti-inflammatory (9) properties of STWs. Thus, the main advantages of the therapeutic use of STWs lies in the fact that these provide a non-aggressive treatment, without considerable side effects, and which also has preventive properties (6, 8, 10, 11). Nevertheless, knowledge associated with the clinical properties of STWs in the context of respiratory diseases is mainly empiric, acquired over a period of centuries, since few well-designed clinical studies exist. Furthermore, although there are some very interesting *in vitro* studies on the effects of STWs on cells of the immune system, such studies are scant. Hence, the question of how exactly these waters modulate the observed clinical amelioration is poorly understood.

In this non-systematic review, we will analyze recent and past data obtained from a number of studies that have contributed toward the elucidation of the mechanisms of action of STWs on the lung epithelia-immune interface. To do that, we have performed a compilation of PubMed publications combining the following search terms “sulfurous thermal waters,” and “hydrogen sulfide” with the terms “allergic rhinitis,” “asthma,” “chronic obstructive pulmonary disease” (COPD) “lung,” and ”lung endothelial cells” with Boolean operator “AND” and “OR.” Various combinations were used, in order to focus on specific search questions of the various analyzed topics. No limitations were made in duration of the study or the demographic data of subjects. Literature published in the last 30 years was included. The inclusion criteria in this review were studies conducted with STW, H_2_S-enriched waters, or H_2_S for airway diseases, allergic, chronic, rhinosinusitis, COPD, or biological targets and effects of H_2_S.

The following outcome parameters examined were included in this review: mucocilliary clearance time, nasal respiratory flow and resistance, adverse effects, and immunoglobulin values.

Overall, in the different searches performed, 7,345 studies were obtained. Of these, in total, 7,114 studies were excluded by reading the title or the abstract since they were not relevant or focused on cellular features or disease-related aspects that were not related to the topic of this review. Of the remaining 231 studies, 59 were excluded from analysis because of redundant information of lower quality than that in included studies or because the work focused on issues that were not fully relevant.

## Thermal Waters: Composition and Biochemical Properties

Depending on their geographical localization, STWs present different physicochemical characteristics. These differences are due to their diverse chemical composition and to the presence of varying amounts of ions and salts, resulting in different therapeutic indications (2, 12).

Portugal is a country with abundant natural mineral (thermal) waters from north to south, as well as in the Portuguese islands, and the frequent visits to bath spas are quite common by the Portuguese population. Among the different types of thermal waters, sulfurous ones are the most common in the north and center of Portugal, with Termas das Caldas da Felgueira (13), Termas de São Pedro do Sul (14), Ferreira et al. (15), and Termas de Unhais da Serra (16) being some of the more representative ones in terms of sulfur-predominant waters. These thermal waters are alkaline (pH = 8.4–8.9), poorly mineralized, and are indicated for the treatment of respiratory, circulatory, digestive, rheumatic and musculoskeletal, as well as metabolic-endocrine diseases (12).

Concerning respiratory diseases, the therapeutic exposure to STWs is performed mostly through inhalation (6, 11, 17), and recently, significant clinical efficacy (e.g., nasal resistance and nasal flow improvement, and reduction of mucocilliary clearance time) was demonstrated when adult and elderly patients underwent hydrogen sulfide (H_2_S)-enriched nasal water inhalations (18). Inhalator exposure efficacy depends upon various aspects which may affect particle deposition in the airways. These include adopted nebulizer, particle size, airway caliber, and patient's breathing pattern. For instance, the nebulizer must be able to produce particles with a diameter <3 μm in order to reach the bronchioli (11). Side effects are another aspect that must always be taken into consideration, whatever the implemented therapy. Even though STWs are generally well-accepted as a safe therapeutic tool due to their low number of side effects (10), these can still occur. In a systematic review and meta-analysis, Keller et al. (19), analyzed all side effects occurring in the pooled total patient population that took part in 13 clinical studies. Focusing on sulfurous waters, after 90 days of STWs treatment, only 19 out of 370 patients presented some adverse events. From those, 13 experienced mild nasal irritations and a sensation of burning, 5 suffered from very limited epistaxis, and one from dermatological hypersensitivity. Moreover, it is of note that even when subjects presented those effects, most of them were local and reversible (19). However, despite common use STWs to achieve a state of well-being and disease amelioration, the cellular and molecular bases underlying these beneficial effects remain unclear.

It was recently found that STWs can induce the production of moderate amounts of neutrophil-attracting chemokines, and low levels of tumor necrosis factor α and interleukin (IL)-6 (9). Even though pro-inflammatory mediators are frequently linked to detrimental situations, moderate inflammatory stress may be regarded as positive, according to hormesis theory (20). Thus, mild stress can stimulate body systems repair, in order to prevent further and more severe damage, provided that this state does not involve the accumulation of irreversible changes (20). In addition, with time, inflammation may change the composition of nasal, sinusal, and lung bacterial flora, which may be associated with the development of resistant strains of bacteria (21). This means that inadequately controlled inflammation may lead to bacterial superinfection. Thus, for subjects with inflammatory respiratory disease, which is stable or in its initial phases, STWs may be a good complement to drug therapy since they may contribute toward prevention of recurrent infections caused by various bacteria (11) and viruses (22), and subsequently avoid the progression to a chronic state. Additionally, thermal waters might be also a useful tool in post-operative recovery in cases of chronic rhinosinusitis with or without polyposis, as observed by Staffieri et al. (23). These authors detected a significant reduction of the numbers of inflammatory cells (particularly eosinophils and mast cells) in the nasal mucosa of patients who were treated with sulfurous-arsenical-ferruginous thermal water inhalation after 6 months of having undergone endoscopic sinus surgery (23). Hence, by playing a preventive role in the progression of inflammatory states, STWs may have a prophylactic effect against further inflammation or subsequent superinfection. Furthermore, as a prophylactic agent, STWs may also reduce or even avoid additional hospital costs and degradation of patients' quality of life which are associated with events, such as frequent infection-induced exacerbations or prolonged hospitalizations. Thus, either as prophylactic or therapeutic tools, STWs may not only improve patients' general health and disease-related clinical parameters, but may also show other benefits from a social and financial point of view (e.g., reduction of drug-related costs, decreased hospitalization and disease-specific healthcare costs, national health care decongestion, and a decrease in school and work absenteeism), although some of these benefits were shown in rheumatological diseases and still need to be addressed in cost-effectiveness studies of STWs treatments for respiratory diseases (see Table 1).

**Table 1:** Major beneficial effects induced by sulfurous thermal waters.

		**References**
Prophylactic	• Preventive role in the progression of inflammatory states or subsequent superinfection	(11, 22, 23)
Therapeutic	• Nasal resistance and flow amelioration • Reduction of mucocilliary clearance time • Reduction of inflammatory influx • Reduction of drug intake	(18, 19) (19) (23, 24) (18, 19)
Socio-economic	• Reduction of school and work absenteeism • Decrease of hospitalization and other disease-related healthcare costs	• Possible in respiratory diseases, but still needs to be addressed in cost-effectiveness studies

*STW inhalation can have either a prophylactic or a therapeutic role, which may be associated with potential socio-economic benefits*.

In thermal waters H_2_S, hydrosulfide ion, and sulfide anion are the most common sulfide species present, with H_2_S being the most abundant (7).

## Biological Properties of Hydrogen Sulfide

Due to its inflammable and corrosive nature, H_2_S was thought to be a poisonous gas. However, in the last decades, it has been reported, along with nitric oxide and carbon monoxide, as a gaseous signaling molecule (25). Indeed, in contrast to nitric oxide, H_2_S is relatively stable in body fluids, appearing as a promising therapeutic agent in several diseases. Nevertheless, it is important to take into account that H_2_S can easily pass from water to air (26) and it is a gas which is soluble in water as well as in physiological liquids, and which is volatilized and broken down *in vivo* (namely in the lungs due to the abundant presence of oxygen) (27). Thus, H_2_S frequently mentioned in studies may indicate a mix of H_2_S and hydrosulfide and sulfide species, alongside with their effects (25, 28, 29).

**Figure 1:**
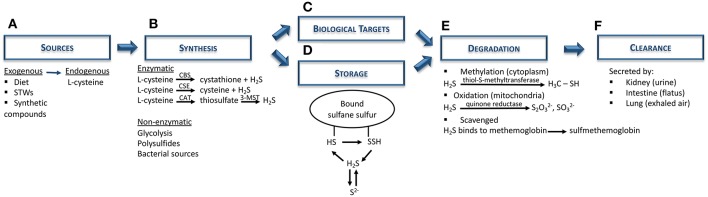
Hydrogen sulfide metabolism: synthesis, storage, degradation, and clearance. **(A)** Both exogenous (diet; sulfurous thermal waters; synthetic compounds, e.g. NaHS) and endogenous (L-cysteine) are the main sources of H_2_S biosynthesis. **(B)** H_2_S is synthesized mainly via CBS and CSE enzymes, although it can also be produced through the combination of CAT and 3-MST. Nevertheless, non-enzymatic sources (e.g., polysulfides, bacterial sources) can also be H_2_S sources. **(C)** Subsequently, H_2_S can either act on its biological targets or **(D)** be stored as bound sulfane sulfur and acid-labile sulfur pools. **(E)** In order to maintain H_2_S levels balanced, this gas undergoes further degradation via oxidation in mitochondria (quinone reductase) or, methylation in the cytoplasm (thiol-S-methyltransferase), or it can be scavenged by binding to hemoglobin. **(F)** Finally, H_2_S is excreted by the kidney (urine), intestine (flatus) or lung (exhaled air). CAT, cysteine aminotransferase; CBS, cystathionine β-synthase; CSE, cystathionine γ-lyase; H_2_S, hydrogen sulfide; HS, hydrosulfide; 3-MST, 3-mercaptopyruvate sulfurtransferase; NaHS, sodium hydrosulfide; S^2−^, acid-labile sulfur; SSH, hydrodisulfide; STWs, sulfurous thermal waters.

H_2_S is a colorless gas with potent reducing properties resulting from geothermal activities (sulfurous mineral water and volcanoes) and found in vegetable proteins (broccoli, garlic), and synthetic compounds (NaHS, GYY4137) (28, 30–34) (Figure 1A). In the human body, H_2_S is produced by a variety of cells (e.g., epithelial, vascular, and smooth muscle cells), and is mainly synthesized from L-cysteine via cytoplasmic and mitochondrial cystathionine β-synthase (CBS) and cystathionine γ-lyase (CSE) enzymes (30, 35). Endogenous H_2_S is also generated by the combined action of cysteine aminotransferase, localized in the cytosol and 3-mercaptopyruvate sulfurtransferase, present in the mitochondria (28, 30, 34, 36). Apart from these enzymatic pathways, H_2_S can also derive from indirect or secondary endogenous sources, particularly from glucose (via glycolysis), glutathione (GSH), inorganic and organic polysulfides, elemental sulfur, and even from bacterial sources present within the gastrointestinal and pulmonary tracts (29, 31, 33) (Figure 1B). After H_2_S synthesis, it can either act on its biological targets or be stored as a bound sulfane sulfur pool, through oxidative formation of hydrodisulfides/persulfides, hydropolysulfides, and polysulfides) (33, 37) as well as an acid-labile sulfur pool, through metal-sulfur clusters, free hydrosulfide ion, and persulfides (29, 34) (Figures 1C,D). In the presence of oxygen, H_2_S undergoes oxidation in the mitochondria by sulfide quinone reductase and also via methylation in the cytoplasm, through thiol-S-methyltransferase (30, 38) (Figure 1E). In addition, free H_2_S can be scavenged by methemoglobin and molecules with metal or disulfide bonds (Figure 1E), and excreted together with biological fluids (urine and flatus), as well as exhaled in breath (Figure 1F) (30, 35).

Due to its high ability to diffuse across lipid membranes without the need of a transporter, H_2_S can easily reach its molecular targets in a variety of cells, including those in respiratory, cardiovascular, and neuronal systems (25, 28, 35, 39, 40). Its stability, storage, and release depend upon pH, temperature, and oxygen concentration of the environment. Thus, at physiological pH only a third of total sulfur amount is in the H_2_S form. At acidic pH, however, H_2_S is the only form present. In contrast, at an alkaline pH only the bisulfide form exists (28). Oxygen also influences H_2_S stability since the presence of oxygen induces its conversion to sulfur, which can be further oxidized to hyposulfite, sulfites, and sulfates. In other words, under aerobic conditions H_2_S is consumed and consequently its effective concentration decreases (29, 41). These findings corroborate the hypothesis that H_2_S may act as a cellular oxygen sensor (42). Indeed, the decrease in H_2_S oxidation under hypoxic conditions has been associated with the production of significant amounts of H_2_S by airway epithelial cells (AECs) (43), akin to production via reduction of preexisting sulfites in mitochondria (44). Fu et al. suggested that this occurs as a result of CSE enzyme translocation to mitochondria, thereby allowing H_2_S synthesis even after a stress-inducing stimulus, such as hypoxia (45). However, a significant increment of mitochondrial H_2_S levels and a decrease of its clearance can, therefore, lead to harmful effects, including vasoconstriction and pro-apoptotic effects (46).

The measurement of H_2_S has turned out to be of major importance to ascertain its putative role in a number of diseases. Although various methods have been used for measuring H_2_S levels in blood and plasma/serum, such as the methylene blue method, sulfite-sensitive ion selective electrodes, and others, these methods have provided discrepant results, with H_2_S levels in plasma ranging between 1 and 100 μM. This high variability has been attributed to the limitation and lack of sensitivity of the techniques (29, 47), to its capacity to diffuse through cellular membranes, and to the extremely short H_2_S half-time *in vivo* (48). Hence, to overcome this obstacle it is mandatory to perform precise biological measurements of H_2_S.

Regarding the airways, different approaches are used to quantify H_2_S levels. On the one hand, Saito et al. have suggested that sputum H_2_S may be a better biomarker than serum H_2_S in lung-related diseases, since its serum levels may reflect other diseases of peripheral organs (49). Moreover, induced sputum was shown to be an effective sample to assess and identify asthma subtypes (50). However, Saito et al. found a negative correlation between H_2_S levels in sputum and serum in COPD patients with acute exacerbations (51). This led the authors to propose that an increase in H_2_S levels in sputum may reflect sequestration of H_2_S from the circulation into the lungs, with the sputum-to-serum H_2_S ratio being a good predictor of exacerbations (51). On the other hand, the measurement of exhaled H_2_S is a non-invasive technique that is not affected by oral conditions (52) and which has been shown to positively correlate with the lung function, particularly forced expiratory volume in 1s (53). Nevertheless, unlike exhaled nitric oxide, H_2_S levels in exhaled air are not used as an H_2_S metabolism detector (54). Therefore, either exhaled or sputum H_2_S could become promising and accurate indicators of airway diseases and airway disease phenotypes.

### Biological Targets of Hydrogen Sulfide

**Figure 2:**
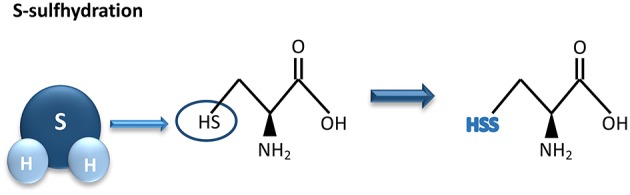
Hydrogen sulfide-mediated S-sulfhydration reaction. H_2_S adds a sulfhydril group to thiol groups of reactive cysteine residues, resulting in sulfhydration of its biological targets. H_2_S, hydrogen sulfide.

Most of the biological activities of H_2_S are exerted through protein S-sulfhydration, a post-translational modification process whereas an H_2_S-derived sulfur group (sulfhydryl) is added to the thiol groups of reactive cysteine residues, originating hydropersulfide (35, 55) (Figure 2).

This formed group enhances reactivity of the protein and may be associated with an increment of its biological activity (55). The degree of protein S-sulfhydration can be influenced by cell pH as well as by the distance between the target amino acids and the active core of the protein (35). Unlike S-sulfhydration, S-nitrosylation caused by nitric oxide results in a reduction of cysteine reactivity. As an example, through S-sulfhydration the endothelial nitric oxide synthase (eNOS) activity increases, as a consequence of eNOS dimerization. In contrast, S-nitrosylation of eNOS induces the formation of eNOS monomers, leading to a reduction of its activity (56).

Thus, H_2_S is regarded as a gaseous signaling molecule that targets a number of important biological processes and pathways, and in this sense contributes to the maintenance of body homeostasis (35, 57, 58). The biological targets include ion channels, second messengers, signaling molecules, and transcriptions factors. Nevertheless, some of the observed H_2_S effects are contradictory and probably result from different cell types and concentrations used.

#### Ion Channels

H_2_S is able to interact with many ion channels and, as a consequence, alter their activity by inducing or inactivating their action. It has been reported that by sulfhydrating ATP-activated potassium channels (${\text{K}}_{\text{ATP}}^{+}$) in smooth muscle cells (SMCs) (35, 59, 60) and small to medium calcium-dependent potassium channels in vascular endothelial cells (61), H_2_S can induce the opening of these channels, thereby allowing K^+^ ion influx, which leads to vasorelaxation.

**Figure 3:**
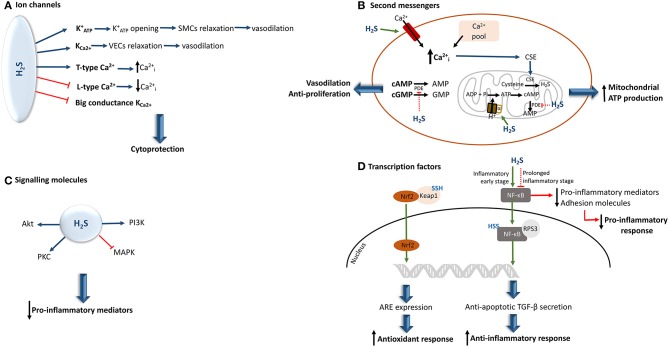
Hydrogen sulfide interaction and effects upon the activity of its biological targets. **(A)** In a physiological concentrations, H_2_S can either activate or inhibit different ion channels, providing a cytoprotective effect to the cell. **(B)** By inducing an increment of intracellular calcium, H_2_S induces the translocation of CSE to the mitochondria, resulting in the generation of H_2_S. In parallel, the H_2_S-mediated S-sulfhydration of the mitochondrial alpha subunit of ATP synthase, allows the augmentation of cAMP levels. This results in the increase of mitochondrial ATP production. Moreover, H_2_S can also inhibit PDE activity, allowing the increase of cAMP and cGMP net levels, thereby conferring vasodilatory and anti-proliferative properties **(C)** If, on the one hand, H_2_S seems to be able to prevent MAPK activation, on the other hand it increases the levels of other kinases, including PI3K/Akt, and protein kinase C, some of which can inhibit the production of pro-inflammatory cytokines. **(D)** The H_2_S-induced S-sulfhydration of Keap 1 induces the translocation of Nrf2 to the nucleus leading to the expression of ARE, which results in the augmentation of antioxidant responses. Moreover, H_2_S can induce NF-κB activation or inhibition. This depends upon the stage of underlying inflammation. ARE, antioxidant response elements; ${\text{Ca}}_{\text{i}}^{2+}$, intracellular calcium; CSE, cystathionine γ-lyase; ${\text{K}}_{\text{ATP}}^{+}$, ATP-activated potassium channels; Keap 1, Kelch-like ECH-associated protein 1; MAPK, mitogen-activated protein kinase; NF-kB, nuclear factor-kappa B; Nrf2, nuclear factor erythroid-related factor 2; PI3K, phosphoinositide 3-kinase; PDE, phosphodiesterase; PKB, protein kinase B; PKC, protein kinase C; SMCs, smooth muscle cells; TGF-β, transforming growth factor-β; SSH, S-sulfhydration; VECs, vascular endothelial cells; the green arrow indicates that the pathway is stimulated and the red arrow indicates that the pathway is inhibited.

In contrast, this molecule has the ability to inhibit big conductance calcium-activated potassium channels (35, 57, 61). Moreover, H_2_S also exerts a regulatory effect on intracellular calcium levels by stimulating or inhibiting the T/L-type calcium channels and in turn may up- or down-regulate several calcium-dependent signaling pathways and enzymes, depending upon cell type (35, 61, 62). Furthermore, through the reduction of inositol-1,4,5-triphosphate receptor present in airway SMCs, H_2_S can also affect calcium efflux in cells (62) (Figure 3A).

#### Second Messengers

Second messengers are important intracellular signaling molecules involved in several cellular pathways and their availability can be affected by H_2_S in a direct or indirect way.

Bucci et al. have observed that H_2_S has the ability to suppress the degradation of cyclic nucleotides by inhibiting the activity of phosphodiesterase, an enzyme that is responsible for their conversion into AMP and GMP (63). Thus, cAMP and cGMP availability increases, making these molecules available for intracellular signal transduction pathways that they are involved in.

The interaction of H_2_S with calcium ion channels directly affects calcium availability in cells. Therefore, the interaction of this gaseous signaling molecule with calcium ion channels and intracellular calcium pools, can lead to a rise in intracellular calcium by inducing its influx and release, respectively (35). This subsequently induces endothelial proliferation. In parallel, the enhancement of calcium levels can also prompt the translocation of CSE from cytosol to mitochondria, thereby stimulating H_2_S synthesis inside this organelle, and subsequent mitochondrial ATP production (45). On the other hand, the decrease in intracellular calcium can also be induced by H_2_S via inhibition of calcium channels, and in turn this suppression seems to be involved in the induction of airway smooth muscle relaxation (64). Moreover, L-type calcium channel inhibition by H_2_S induces membrane permeability and, subsequently, decreases elastase release (65) (Figure 3B).

Recently, it was reported that, under pathological conditions, the S-sulfhydration of the alpha subunit of ATP synthase supports its activation. In this manner, mitochondrial bioenergetics is maintained (66).

#### Signaling Molecules

Signaling molecules act as signal transmitters allowing the cross-talk between cells. H_2_S has also been reported as capable of modulating the activity of several signaling molecules involved in biological processes, including phosphorylation, oxidation and degradation of proteins.

Thus, H_2_S has been shown to regulate the activity of the mitogen-activated protein kinase (MAPK). In primary human lung endothelial cells, exogenous H_2_S was able to prevent MAPK activation (67). H_2_S has also been reported to increase the levels of other kinases, including PI3K/Akt, and protein kinase C, some of which can inhibit the production of pro-inflammatory cytokines (48, 57, 67–69) (Figure 3C).

#### Transcription Factors

Transcription factors are proteins responsible for regulating genetic information transcription and can also be a target for H_2_S action. Thus, by undergoing S-sulfhydration transcription factors can induce up- or downregulation of gene expression.

The inactivation of nuclear factor-kappa B (NF-κB) through S-sulfhydration, blocks its translocation to the nucleus, which leads to the suppression of pro-inflammatory mediators and production of adhesion molecules (40). Nonetheless, depending upon the specific inflammatory stage, an opposite effect can be observed (70). Thus, in an initial phase of the inflammatory response, pro-inflammatory cytokine tumor necrosis factor α (TNF-α) can induce CSE transcription, thereby enhancing H_2_S synthesis. Hence, the newly generated H_2_S sulfhydrates the p65 subunit of NF-kB, promoting its binding to the coactivator ribosomal protein S3, which results in an increment of anti-inflammatory cytokine transforming growth factor-β secretion as well as the stimulation of anti-apoptotic transcriptional activity (57, 70). However, it should be highlighted that in both situations the inflammatory response is diminished, and consequently, oxidative stress decreases.

Moreover, as a consequence of S-sulfhydration of Kelch-like ECH-associated protein 1 (a negative regulator of factor erythroid-related factor 2 activity), the nuclear factor erythroid-related factor 2 is then translocated to the nucleus. This results in the expression of antioxidant response elements (68, 71) (Figure 3D).

Consequently, S-sulfhydration appears to play an important role in protecting cellular damage, since it has vital implications in anti-inflammatory as well as in antioxidant defenses.

### Biological Effects of Hydrogen Sulfide in the Lung and Associated Immune System Cells

The respiratory mucosal epithelium is the first internal line of defense by acting as a major physical barrier between internal and external environments. The airway surface liquid (comprising mucus and periciliary liquid layers) covers this epithelial surface made up of goblet cells, ciliated cells, basal cells, and club–Clara cells (72–74). It is produced by the epithelial cells, which are also in contact with respiratory gases, thereby constituting the air-liquid interface (75). A connective tissue fibroblast-rich layer is located underneath the epithelial surface, and is involved in the maintenance of tissue homeostasis and wound healing (76). This cell layer also contains a variety of cell types, including cells of the innate and adaptive immune system (76). In this layer is located another lung barrier formed by endothelial cells, which separate the bloodstream and vessel walls. The endothelium also has other functions (e.g., blood and oxygen supply, nutrient delivery, and immune cell trafficking) (75, 76).

In physiological conditions, H_2_S-related enzymes are expressed in the human lungs, namely in SMCs and primary fibroblasts (60, 77). It is now acknowledged that H_2_S is required for the development of lung vasculature and alveolarisation (78), and in other lung functions, including airway tone and pulmonary circulation (30). Furthermore, H_2_S appears to be involved in various processes namely airway mucolytic activity, oxidative stress, inflammatory state, cell proliferation, and apoptosis (10, 30). These will be briefly reviewed below (see also Table 2).

**Table 2:** General biological effects of hydrogen sulfide at physiological levels in the lungs.

**Biological effects**		**Model of study**	**Disease**	**Thermal water or H_**2**_S donor/control**	**Application**	**References**
Mucolytic	Less viscous mucus	Human patients	Chronic inflammatory processes	Sulfur-chloride-bicarbonate-alkaline/-	12-days 1x daily humid-hot inhalation for 10 min	(10)
	Reduction of mucociliary transport time	Human patients	Chronic sinonasal disease	Sulfurous-arsenical-ferruginous/ISCS	12-days 1x daily warm vapor followed by nasal aerosol	(8)
			Chronic rhinosinusitis	Sulfurous/Physiological solution	12-day 1x daily warm vapor inhalations and nasal irrigations	(6)
			Chronic sinonasal inflammation	Radioactive water/-	14-days 1x daily warm vapor inhalations followed by nasal aerosol for 10 min	(24)
	Increment of mucociliary clearance	*In vitro* pig tracheae and cultured H441 cell line	Hypoxia	Na_2_S/Hyperoxia (100% O_2_)	100 μM Na_2_S applied to the apical compartment	(43)
Antioxidant	Increment of GSH availability and SOD levels	*In vivo* female BALB/C mice sensitized with ovalbumin (OVA)	Inflammatory lung diseases	NaHS/iNOS inhibitor	Treatment 30 min before each OVA challenge during 6 days	(79)
Anti-inflammatory	Suppression of leukocyte adherence and migration	*In vivo* male C57BL6/J mice 8 weeks of age	Myocardial ischemia-reperfusion injury	Na_2_S/-	NA_2_S administered into the left ventricular at the time of reperfusion at different doses	(80)
		*In vivo* rats	Acute inflammation	NaHS and Na_2_S/-		(81)
	Inhibition of macrophage inflammation	*In vitro* THP-1 Monocytes and RAW Macrophages cell lines	Macrophage inflammation	NaHS/Hexyl acrylate	Pre-treatment with NaHS for 30 min	(82)
	Inhibition of myeloperoxidase activity	*In vitro* inflamed colon cells from male Wistar rats and isolated neutrophils from human blood	Colitis and healthy state	Na2S/-	Interaction of sulfide and MPO assessed using several spectroscopic techniques	(83)
Antiviral/antibacterial	Protective role in controlling viral assembly/release	*In vivo* 10- to 12-week old BALB/c mice or C57BL/6J mice genetically deficient in the CSE enzyme	RSV infection	GYY4137/-	GYY4137 administration performed i.n. at different doses and timing of RSV infection	(22)
		*In vitro* A549 cell line and SAE cells	RSV infection	GYY4137/-	GYY4137 administrated either prior to infection, but not throughout the duration of infection, or at different times p.i. after the viral inoculum was removed	(84)
	Inhibition of bacterial biofilm production	Human patients	RURT infection	Sulfurous/physiological solution	12-day 1x daily warm vapor inhalations	(11)
Analgesic	Induction of endogenous opioid system activation	*In vivo* male Wistar rats	Visceral pain	Na_2_S/vehicle	100 μmol/kg Na_2_S administered 5 min before CRD	(85)
Anti-proliferative	Inhibition of SMCs proliferation	*In vitro* airway SMCs from donor lungs	–	NaHS or GYY4137/-	100 μM H_2_S donors exposure for 2 or 3 days	(32)
Anticancer	Increase in the production of metabolic acid lactase of cancer cells	*In vitro* MCF10A, MCF7, WI38, and HepG2 cell lines	Cancer	NaHS or GYY4137/ZYJ1122	400 μM H_2_S donors exposure for 5 days	(86)
	Impairment of pH regulatory system of cancer cells					

*A549 cell line, human alveolar type II-like epitelial cell line; CRD, colorectal distension; GYY4137, morpholin-4-ium 4 methoxyphenyl [morpholino] phosphinodithioate, a novel water-soluble, slow releasing H_2_S compound; GSH, glutathione; H_2_S, hydrogen sulfide; H441, human lung epitelial cell monolayers; HepG2, human hepatocelular carcinoma; ISCS, isotonic sodium chloride solutioN; i.n., infected intranasally; MCF10A, human breast adenocarcinoma; MCF7, human breast epihelial; MPO, myeloperoxidase; Na_2_S, sulfur salt; NaHS, sodium hydrosulfide; p.i., post-infection; RURT, recurrent upper respiratory tract infections; RSV, Respiratory syncytial vírus; SMCs, smooth muscle cells; SOD, superoxide dismutase; SAE cells, small alveolar epitelial cells; WI38, normal human lung fibroblast; ZYJ1122, structural analog of GYY4137, which does not release H_2_S*.

*Exposure to H_2_S at physiological levels appears to have beneficial effects in various pulmonary processes, namely airway mucolytic activity, oxidative stress, inflammatory state, viral and bacterial infection, pain relief, cell proliferation, and carcinogenic*.

#### Mucolytic Effect

Mucins are secreted by goblet cells, submucosal glands in upper airways (75, 87), and alveolar cells in lower airways (74). By secreting mucins, the mucus traps and absorbs potential harmful pathogens and irritants which are subsequently removed from the respiratory tract by the process of ciliary beating which mediates appropriate mucociliary clearance (73). An effective removal of mucus protects the respiratory epithelium, acting as a vital role in airways homeostasis (88). Both endogenous and exogenous H_2_S show positive effects upon the respiratory tract by modulating the mucolytic activity. This appears to result from the interactions between H_2_S and the disulfide bonds of mucins, resulting in breakage of the latter, which allows the mucus to become less viscous (10). The production of endogenous H_2_S induces the opening of ${\text{K}}_{\text{ATP}}^{+}$ and the activation of the cAMP pathway (30). Additionally, exogenous H_2_S inhibits Na^+^/K^+^-ATPase and calcium-sensitive potassium channels in human bronchiolar epithelia, thereby triggering electrolyte absorption (33, 89).

This leads to an increase in mucociliary clearance, and therefore the elimination of foreign microorganisms can be more effective (43). Krause et al. also proposed that inhibition of transepithelial sodium absorption (via inhibition of Na^+^/K^+^-ATPase) is favored under acute hypoxia, in order to avoid H_2_S degradation, as well as exogenous H_2_S exposure (43). Additionally, an amelioration of mucociliary function was observed with inhalation of exogenous H_2_S, as confirmed by a substantial reduction of mean mucociliary transport time in patients with chronic rhinosinusitis (8, 24).

#### Antioxidant Effect

In a well-controlled environment, reactive oxygen species (ROS) are responsible for cell signaling activation, enhancement of pro-inflammatory cytokines acting as mediators of immunity, and in addition, ROS are also able to protect the cells/tissues. Simultaneously, a balance is achieved with a tight regulation performed by antioxidants (e.g., reduced glutathione, GSH) which directly scavenge those species that are formed, thereby inhibiting any excessive production, by removing or repairing cellular damage or modifications induced by these reactive species.

In the lungs, exposure to H_2_S promotes a boost of antioxidant effects, such as (i) increase in GSH availability, and superoxide dismutase levels (28, 79, 90), and (ii) generation of ATP, replacing oxygen in mitochondrial respiration (91). The reported H_2_S-induced mitochondrial protection related to this antioxidant effect is known to result from mitochondrial cytochrome *c* oxidase inhibition and also from its capacity to modulate cellular respiration, thereby preventing the generation of ROS (48). Consequently, there is an increase in scavenging capacity (30, 34, 92), as well as an induction of endogenous antioxidant defenses (39). Hence, H_2_S antioxidant features seem to involve both an indirect action and an induction of endogenous antioxidant defenses. For instance, by stimulating cysteine and cysteine transporter activity, H_2_S induces an augmentation of substrate levels that are necessary for GSH production (29). Unlike large size antioxidants, H_2_S can easily cross both plasma and mitochondrial membranes. This allows H_2_S to more promptly reach its biological targets, and therefore it is considered to be more effective at diminishing cellular oxidative stresses, and at increasing antioxidant defenses (80). Braga et al. demonstrated that antioxidant effects of STWs, an exogenous H_2_S source, provide protection against oxidative DNA damage (93).

#### Anti-inflammatory Effect

As the first line in contact with foreign species, AECs can act either as a modulator (during homeostasis) or as a sensor (post-injury lung homeostasis) of innate and adaptive immune systems (74). In healthy states, macrophages are the main immune cell type present in the airways, accounting for about 60–70% of all cells. Neutrophils represent 30–40% of total cells, while eosinophils are rare, not exceeding 2% (94). Beneath the epithelial layer, dendritic cells, macrophages, and mast cells can be found in the lamina propria. Dendritic cells and macrophages, in particular, can detect pathogens that have crossed the lung epithelium, phagocytose and destroy by microbicidal mechanisms, thereby contributing toward avoidance of significant infections.

Under a normal state the recognition of pathogen-associated molecular patterns (PAMPs) or damage-associated molecular patterns (DAMPs) occurs through specific membrane or cytosolic receptors present on epithelial cells, and results in the activation of several signaling pathways (e.g., via MAPK, NF-κB, among others) (95–97). Therefore, pro-inflammatory cytokines, chemokines, antimicrobial proteins, and antiviral substances are produced and released by AECs, which allows them to have a crucial role in the recruitment and activation of innate (e.g., macrophages, eosinophils, dendritic cells) and adaptive (e.g., T and B cells) immune cells (96, 97). The presence of these cells allows several responses (phagocytosis, dendritic cell maturation, chemotaxis, inflammasome activation, and others) to be triggered (97). Subsequently, cells of the adaptive immune system (e.g., T and B cells) are activated. Thus, when homeostasis is affected, the usual function of AECs is reduced and pro-inflammatory activities increase significantly.

The anti-inflammatory properties of H_2_S can in part be explained by its potent reducing, antioxidant, and scavenging features. Nevertheless, there are controversial results concerning H_2_S properties, since it appears to exert both pro- and anti-inflammatory effects. Some authors have shown an *in vitro* promotion of granulocyte survival via H_2_S-induced inhibition of caspase-3-cleavage and p38 phosphorylation (98, 99) and in an oxidative stress environment, activated neutrophils seem to be able to convert H_2_S into sulfite, which is associated with inflammation (100). Moreover, there are studies suggesting that this gaseous transmitter is involved in GSH depletion and ROS formation (101), and consequently induction of mitochondrial cell death pathways (102). Meanwhile, others have suggested the participation of H_2_S in some key anti-inflammatory pathways, such as: (i) suppression of leukocyte adherence and migration, mediated by ${\text{K}}_{\text{ATP}}^{+}$ activation in endothelial cells and leukocytes (81), (ii) inhibition of oxidized low-density lipoprotein-induced macrophage inflammation via NF-κB suppression (82), leading to a reduction of several pro-inflammatory cytokines (e.g., IL-1β, IL-6, and IL-8) (103–105), and (iii) reduction of neutrophil toxic effects by inhibiting myeloperoxidase activity (83). However, these discrepancies may be related to differences in H_2_S concentration. In fact, beneficial effects of H_2_S generally prevail at lower concentrations, whereas deleterious effects are observed at higher levels and at fast-releasing rates, which is comparable to effects seen with carbon monoxide and cyanide (28, 34, 48). In addition, inhalation of H_2_S at high acute levels or chronic lower-level exposure can also induce lung inflammation and toxicity (48).

Nonetheless, it has been known for some time that STWs can exert a direct anti-inflammatory effect in the lung. Exposure to these natural mineral waters may induce increased release of IL-10 levels by *in vitro* exposed primary human monocytes (9). Furthermore, an increase in the levels of IL-10 in saliva were also described in patients who had been treated with STWs (9). Moreover, STWs can simultaneously up-regulate immunoglobulin (Ig) A (an anti-inflammatory immunoglobulin) levels, as well as down-regulate the levels of IgE (a pro-inflammatory immunoglobulin) and cytokines secreted by eosinophils (90). This suggests that by stimulating anti-inflammatory defenses, STWs decrease the formation of ROS, and therefore modulate the pro-inflammatory state.

#### Antiviral and Antibacterial Effects

In several pulmonary diseases (asthma, COPD, among others) recurrent respiratory tract infections may occur due to a fragile protective mechanism system, which may enhance a pro-inflammatory state (106). As these diseases progress, viral and bacterial infections may become more frequent, and increasingly more difficult to treat. Consequently, the patients' health state worsens and their quality of life decreases.

Using AECs, Chen et al. shown a link between CSE inhibition and a significant increase in viral replication and chemokine secretion, which are reduced when these cells are exposed to a slow-releasing H_2_S donor (107). Serum H_2_S levels are significantly decreased in COPD subjects with very symptomatic exacerbations induced by bacteria and viruses (Type I; Anthonisen criteria), compared to control subjects. However, when COPD exacerbations are less symptomatic (Type II or III; Anthonisen criteria), serum H_2_S levels are higher than those in control subjects (107). Moreover, when COPD patients who exacerbated are treated with antibiotics, serum H_2_S levels are lower than in those patients who did not require antibiotic treatment. Taken together, these results indicate that endogenous H_2_S synthesis is induced in order to counter the infections-mediated exacerbations (107). Currently, the most frequent therapies used to treat lung viral and bacterial infections are antiviral and antibiotics. However, over time, antibiotic-resistant bacteria may be increasingly observed.

To overcome these problems, some studies focused on the clinical efficacy of therapeutic S-based compounds (e.g., H_2_S donors, STWs) (22, 84). Previous data showed that these compounds exhibit a protective role as an antiviral agent since they were able to control viral assembly/release (84). In turn, an improvement in viral infection-induced airway hyperresponsiveness can be prompt and may be associated with a decrease in the expression of pro-inflammatory mediators as well as in inflammatory cell influx into the lung (22). Furthermore, a study where patients with chronic sinonasal disease inhaled STWs showed a significant nasal flow improvement in these patients, which was associated with lowering the numbers of nasal bacteria (8). Also, a study in children with frequent upper respiratory infections showed an important reduction in frequency, duration, severity, and social impact of the infectious episodes with STWs treatment (11). This antibacterial effect may be due to H_2_S toxicity since only sulfur-bacteria and some microorganisms survive in the presence of S-based compounds, such as those present in STWs (108). This anti-pathogenic role also seems to be linked to the inhibition of bacterial biofilm production, as a result of blockage of the synthesis of microbial adhesins, thereby interfering with bacterial adherence to epithelia and reducing pro-inflammatory potential (6). Also, Varrichio et al. observed a significant reduction of bacteria in children with respiratory infections treated with salso-sulfide thermal water (17). In this context, Benedetti et al. proposed NF-kB pathway as a target of H_2_S donors to reduce bacteria-induced inflammation (40).

Thus, S-based compounds can be a good complementary approach to conventional drug therapy, thereby increasing clinical efficacy of these treatments.

#### Analgesic Effect

Whether and how exactly H_2_S can modulate an anti-nociception effect remains to be clarified. In patients with perennial allergic rhinitis (AR), a previous study involving inhalation of heated water showed that heat therapy may contribute to relief of perinasal pain and can somehow prevent nasal congestion (109). However, in this study, the heated water was not described in terms of H_2_S content. Nevertheless, hyperthermia acts as a stimulus of neuroendocrine responses, with an increase in opioid (β-endorphin and Met-encephalin) and adrenocorticotropic hormone levels (110, 111). As mentioned previously, the main compound present in STWs is H_2_S, and since this therapy is usually performed through inhalation at a temperature between 36 and 38°C (13, 14, 16), STWs therapy can somehow mediate an analgesic effect in respiratory diseases, mainly in the upper airways, during treatment. This can be explained in part, by the high temperatures of STWs but also by the role of H_2_S in the activation of ${\text{K}}_{\text{ATP}}^{+}$. In fact, some studies have suggested that the H_2_S-mediated analgesic effect is based upon the opening of neuronal ${\text{K}}_{\text{ATP}}^{+}$, which in turn is related with endogenous opioid system activation (85, 112).

#### Anti-proliferative Role

In the lung, cAMP and cGMP are responsible for mediating endothelium-dependent dilatation, since they are involved in lung vascular homeostasis. In fact, a reduction in their levels may lead to pulmonary hypertension, a disease characterized by high blood pressure that affects the lungs' arteries which become narrowed, blocked or destroyed (113). Since H_2_S inhibits the activity of phosphodiesterase, cGMP net levels increase (63). Therefore, H_2_S might eventually act as a good auxiliary agent in the treatment of pulmonary hypertension by preventing the proliferation of vascular SMCs and consequently promoting vasodilation of lung blood vessels.

Perry et al. proposed that both endogenous and exogenous H_2_S might be able to control airway SMCs proliferation and cytokine release, namely IL-8, by suppressing these cell types. This occurs through inhibition of extracellular signal-regulated kinase 1/2 phosphorylation and extracellular signal-regulated kinase-1/2 p38 MAPK, with CBS, and not CSE, the main enzyme involved in the endogenous H_2_S production (32). Although H_2_S inhibits the proliferation of SMCs, it promotes the growth of endothelial cells (60, 114).

The inhibitory effect upon cell growth induced by exogenous H_2_S was also observed by a reduction in lymphocyte subset proliferation, with a subsequent decrease in T cell-derived IL-2 production (115). Similar results were obtained in an *in vitro* study using STWs (116). This anti-proliferative effect was also shown by Baskar et al. when exogenous H_2_S exposure caused human lung fibroblast cell death by inducing an increase in DNA lesions in these cells (102). In parallel, cell cycle regulators (p53, p21, and Ku proteins) were activated, being responsible for cell death via apoptosis (102). Therefore, the anti-proliferative effect prevents further degradation of DNA and allows the removal of injured cells by a controlled cell death process. In accordance with this, H_2_S has been shown to exert a direct suppression effect on human lung fibroblast migration, proliferation, and transdifferentiation into myofibroblasts (117).

#### Anticancer Effect

During the last decade, some data have suggested various anticancer effects related to H_2_S action (118–121). It was proposed that H_2_S-mediated anticancer role is based upon the combined capacity of H_2_S to increase the production of metabolic acid lactase and to impair pH regulatory system of cancer cells (86).

However, in contrast, and despite the anticancer efficacy in various types of cancer cells (e.g., gastric carcinoma, renal tumor), other studies have shown that H_2_S may induce tumor cell proliferation, and migration, as well as angiogenesis, via inhibiting apoptosis (122–124). Jia et al. observed hepatoma cell suppression when CBS/H_2_S pathway was inhibited (125). Moreover, Szczesny et al. have also found that besides normal lung epithelial cells, epithelial lung adenocarcinoma cells also have the ability to synthesize H_2_S, in order to maintain their bioenergetics function and to increase its mitochondrial DNA repair processes. In this sense, cancer cell viability was preserved (126).

Hence, in a cancer situation the type of treatment must be re-assessed since H_2_S seems to be advantageous for cancer cell proliferation. Nevertheless, the H_2_S-induced inhibitory effects on cancer cell proliferation depend not only on H_2_S dose and length of exposure, but also on tumor types. In this context, a continuous and prolonged exposure to low H_2_S concentrations significantly affects cancer cell survival activity (127). In turn, with continuous, high-level exposure to H_2_S, the cell survival rate is severely affected in both cancer and non-cancer cells (128). Oláh et al. have also indicated varying H_2_S levels as an explanation for these inconsistent findings regarding anticancer effect of both H_2_S inhibitors and donors (129). Nevertheless, these authors suggested that if on the one hand optimal endogenous H_2_S concentration may induce proliferation of cancer cells, on the other hand both endogenous H_2_S inhibition (decrease of its levels to below optimal concentration) and exogenous delivery (increment of its levels to above optimal concentration) may suppress cancer cell proliferation (129). Clearly, H_2_S-mediated effects have to be further studied, namely with various cancer cells lines *in vitro* as well as in animal models of various cancers, before any firm conclusions can be drawn.

It should also be taken into account that H_2_S may have a biphasic biological effect, since at low-to-moderate concentrations H_2_S has a physiological role, whereas at higher concentrations, it exerts a more pathological role, as summarized in Table 3.

**Table 3:** Dual biological effects of hydrogen sulfide: beneficial vs. deleterious.

	**Beneficial effects (low H_**2**_S levels)**	**References**	**Deleterious effects (high H_**2**_S levels)**	**References**
Antioxidant/oxidant	• Increase in GSH availability • Scavenging of ROS • Reducing agent	(79, 90) (48) (39, 92)	• Increase in ROS • DNA damage	(101) (100)
Anti-inflammatory/inflammatory	• Suppression of leukocyte adherence and migration • Inhibition of pro-inflammatory genes/cytokines • Increase in anti-inflammatory cytokines	(80, 81) (90)	• Promotion of granulocyte survival • Conversion of H_2_S into sulfite • GSH depletion and consequent ROS formation	(98, 99) (100) (101)
Bioenergetic	• Mitochondrial ATP synthesis • Mitochondrial cytochrome *c* oxidase inhibition	(91) (48)	• Induction of mitochondrial cell death pathways • Reduction of ATP levels • Inhibition of mitochondrial respiration	(102)
Modulation of cancer	• Procarcinogenic	(122–124, 126)	• Anticarcinogenic	(34, 118–120)

*H_2_S, hydrogen sulfide; GSH, glutathione; ROS, reactive oxygen species; ${\text{K}}_{\text{ATP}}^{+}$, ATP-activated potassium channels*.

*Exposure to low levels of is associated with beneficial effects. In contrast, exposure to higher levels and fast-releasing rate of exposure to H_2_S is associated with deleterious effects*.

### Effects of Hydrogen Sulfide in Respiratory Diseases

The human nose and lung are continually exposed to indoor and outdoor agents (e.g., allergens, tobacco smoke, and pathogens). As a result, the airway mucosal epithelium, as an internal line of defense needs to play numerous roles in order to eliminate these foreign agents, thereby allowing normal airway function. On the other hand, after chronic injury and inflammation, the dysregulation of airway epithelial cell function may lead to the pathogenesis of lung diseases, such as AR, asthma, and COPD. These airways diseases are characterized by frequent or persistent respiratory symptoms and intermittent or persistent airflow limitation (130, 131). Moreover, besides the involvement of inflammatory cells and oxidative stress in the pathogenesis of AR, asthma, and COPD, there are various studies suggesting that these patients present alterations in H_2_S metabolism (132–137).

#### Allergic Rhinitis

AR is a chronic inflammatory disease that affects the nasal airways, which become inflamed and engorged after exposure to an allergen to which patients are sensitized. In the nasal mucosa of AR patients, the relevant allergens bind to membrane-bound IgE on mast cells. Cross-linking of various IgE molecules by the allergen induces mast cell activation with rapid release of various pro-inflammatory mediators which are involved in development of acute symptoms. Eosinophils and lymphocytes are also involved, being crucial to the development of chronic symptoms (138, 139). In fact, this inflammatory process disturbs some features of the nasal mucosa, since inflammation is associated with swelling of sinusoidal capacitance vessels, reduction of nasal airway passages size, and an increase in mucus production (140). In addition, nasal airway remodeling may also take place in AR, although whether this really occurs remains controversial. In fact, some studies claim that remodeling is involved, since structural changes (e.g., nasal tissue glandular hypertrophy, collagen or extracellular matrix deposition) were observed, as compared with healthy controls (141, 142). However, in contrast other studies have not found such an association (143, 144).

Park et al. have shown that H_2_S can be found in human nasal mucosa as well as in the plasma of healthy subjects. This may be partially explained by the presence of CBS and CSE in human nasal epithelium (134). While CBS is mainly distributed in the superficial epithelium and submucosal glands, CSE is exclusively localized in vascular endothelium and surrounding smooth muscles (133, 134). In the nasal and sinus mucosa, the amount of H_2_S was shown to be increased in mild and moderate/severe persistent AR (134). In this context, there is an enhancement of human H_2_S-synthesized enzymes, mRNA and protein levels, which consequently leads to a significant increment of H_2_S levels in human airway (134). This may indicate a compensatory mechanism to attempt to revert the pro-inflammatory state.

Although there are few data specifically regarding STWs-treatment of AR, an amelioration in AR patients suffering from allergen-specific non-seasonal rhinitis when treated with a S-based compound water was observed, and this was associated with a reduction in total IgE and an increase in IgA serum levels (145). In accordance with this, it was suggested that STWs may exert an immunomodulatory activity by inducing an increase in IgA levels in nasal mucus (90). Another study also showed significant amelioration when AR patients were treated with STWs, namely in terms of a significant decrease in nasal flow resistance and nasal mucociliary transport time in 84% of subjects (146). Likewise, a significant reduction of IgE and an increase in IgA levels as well as an improvement of subjective symptomatology assessment scale were also observed (146). Data from these studies suggest a compensatory mechanism in order to reduce the presence of pro-inflammatory mediators and an improvement of the inflammatory state. However, clearly more thorough studies on the immunomodulatory and anti-inflammatory effects of STWs treatments in AR patients (as well as in patients with chronic rhinosinusitis) are needed.

Due to common immunopathophysiology, there is a close relationship between AR and asthma (“single airways concept”). In fact, AR is regarded as a risk factor for the development of asthma (147). Thus, although few studies have been carried out with STWs treatment in patients with asthma, we shall now analyse this context.

#### Asthma

Asthma is a heterogeneous disease, usually characterized by chronic airway inflammation. Asthma is thus regarded as a long-term inflammatory disorder that results in excessive smooth muscle contraction, hyper-responsiveness, and variable airflow obstruction and bronchospasm. The development and progression of asthma are associated with airway inflammation (inflammatory cells and cytokines), mitochondrial dysfunction, and oxidative stress (148). Excessive airway mucus production is a feature of asthma, particularly in more severe stages, and mucus may even block the airways, which may lead to death by suffocation (149). The difficulty in controlling asthma is partially explained by its clinical and cellular phenotype heterogeneity. Thus, asthma patients may be grouped into different clusters which are associated with preferential features. In this context, one partial but possible subdivision of patients may be into those who preferentially present an eosinophilic bronchial infiltrate and those that have a preferentially neutrophilic profile (150, 151). In addition, it is fundamental to remember that AECs and SMCs are also some of the major cell types involved in asthma pathology, since they are sources of excessive mucus production and the mediators of cell contraction, respectively. This is supported by the underlying episodes of bronchial smooth muscle contraction in asthma patients (152). Likewise, both AECs and SMCs are responsible for facilitating the amplification of lung inflammation, by recruiting T cells, which contributes to an uncontrolled pro-inflammatory environment. All of these actions are associated with histological changes in the airways (e.g., increased bronchial wall thickness, mucous metaplasia, and smooth muscle hyperplasia and hypertrophy) which may lead to the impairment of pulmonary function (153).

In human studies, a significant decrease in H_2_S serum levels was shown in patients with stable asthma, in comparison with healthy subjects. Such a decrease was even more pronounced in asthmatic patients with severe acute exacerbations. It should be pointed out that a similar type of reduction in H_2_S serum levels was also observed in smokers compared to non-smokers. Moreover, such a decrease was negatively correlated with the percentage of sputum neutrophils in patients with acute asthma (137). Furthermore, both serum H_2_S levels and lung function parameters were found to be decreased in asthmatic children, suggesting a positive correlation between these two parameters (136). In contrast, Saito et al. found elevated H_2_S levels in the sputum of asthmatic subjects, but without significant differences between mild and severe stages (49). These authors also observed a positive correlation between sputum H_2_S levels and sputum neutrophil counts. Nevertheless, serum H_2_S levels presented a negative correlation with sputum macrophage (49) and eosinophil counts (53).

Previous data have also shown that H_2_S donors induce vascular smooth muscle relaxation, while they suppress the proliferation of airway SMCs and IL-8 release in humans (32). Since airway SMCs express high amounts of H_2_S-related enzymes, it appears that the lack of H_2_S may be involved in the progression and worsening of asthmatic airway obstruction.

However, exposure to environmental H_2_S levels in excessive concentrations may have deleterious effects in asthma patients. In an epidemiological study carried out in northeast Nebraska (USA), a positive correlation was observed between the frequency of hospital visits by pediatric and adult asthma patients, due to exacerbations, and high (>30 ppb) outdoor total reduced sulfur and/or general H_2_S levels on the previous day (154). However, it should be noted that actual individual levels of exposure to these sulfur species were not recorded but only inferred. In contrast, Bates et al. did not observe a significant increase in the risk of development of symptoms in asthmatic subjects when exposed to H_2_S from geothermal activity in New Zealand (155). This suggests that, in certain settings, or in prolonged ambient exposure inhalation of high levels of environmental sulfur species may trigger asthma exacerbations, although this must be studied with more robust study designs and compared with intermittent and controlled exposure which is the situation observed in Spas.

#### Chronic Obstructive Pulmonary Disease

COPD is an airways disease caused by significant exposure to noxious particles or gases. The chronic, persistent, airflow limitation associated with COPD is caused by a mixture of small airways disease (e.g., obstructive bronchiolitis) and parenchymal destruction (emphysema) (131). Thus, COPD is characterized by an irreversible persistent decline of airflow associated with an enhanced chronic inflammatory response and emphysematous changes (156). It is well-known that COPD non-smokers and smokers share epithelial susceptibilities features, with direct or indirect cigarette smoking exposure being the main trigger for the development of this disease. In addition, most COPD patients have other chronic co-morbidities, with a proportion of them also having features of asthma–potential Asthma-COPD overlap/ACO (157, 158).

COPD is well-characterized by severe lung structural and functional changes, namely basal, goblet and mucous cell hyperplasia, as well as airway fibrosis (159, 160). Furthermore, a chronic influx of inflammatory cells (T lymphocytes, neutrophils, and alveolar macrophages) in bronchial wall and lumen is also involved in the pathophysiology of the disease (161). As a result of the pro-inflammatory actions, particularly those induced by neutrophil-derived enzymes, the lung parenchyma is destroyed in many cases, leading to emphysema (106, 161, 162).

In a small study performed by Sun et al. using human peripheral lung tissue samples from six patients with COPD, as well as from eleven healthy non-smokers and seven smokers with normal lung function, despite H_2_S levels being quite similar among the groups, a significant decrease in CSE protein levels and an increase in its mRNA levels was observed in COPD patients as well as in smokers (135). However, in contrast, CBS mRNA levels were reduced in COPD patients as well as in smokers, suggesting that H_2_S metabolism may be altered in the lung tissue of COPD patients, just as it is in smokers (135). Moreover, the same group showed a negative correlation between exhaled levels of H_2_S and sputum eosinophilia, in a larger group of 77 COPD patients, suggesting that increased levels of exhaled H2S may predict a non-eosinophilic phenotype in these patients (163). Nevertheless, temporal stability of such phenotypes must be confirmed, since it may vary over time. In fact, Chen et al. also showed that serum H_2_S levels in COPD subjects vary longitudinally, with higher levels being more frequently seen in a stable state than in acute exacerbations of COPD (132). Both in healthy subjects and in COPD patients with acute exacerbations, H_2_S levels were more significantly reduced in smokers than in non-smokers. Also, in this study, regarding serum H_2_S levels, a negative correlation with sputum neutrophils, and a positive association with sputum lymphocytes and macrophages was observed in all patients with COPD. Finally, it seems that H_2_S availability is also related to the stage of airway obstruction in COPD since its levels are significantly decreased in the more advanced stage III than in the milder stage I (132). Thus, the authors justify the enhancement of serum H_2_S levels in stable COPD patients as a compensatory mechanism. Nevertheless, increased levels of H_2_S were observed in COPD subjects with an exacerbation, in comparison with patients with stable COPD, healthy smokers, and non-smokers (51). Despite certain contradictions founded in the previous studies, all indicated significant changes of H_2_S and H_2_S-synthesized enzymes levels in COPD subjects. These H_2_S metabolism alterations seems to contribute, at least in part, to exacerbations and worsening of this respiratory disease state, affecting general lung function.

A significant reduction of oxidative stress and an amelioration of symptoms in subjects suffering from moderate to severe COPD were observed after a 12-day inhalation with STWs and 1 month after the end of the treatment (92). Similar beneficial effects have been observed in patients with chronic rhinosinusitis (6). Further studies focusing on mechanisms underlying such STWs-driven improvement in COPD patients are warranted.

In summary, in spite of generally effective drug-based treatments for most cases of AR, bronchial asthma, and COPD, some patients still show a sub-optimal response to such treatments. Furthermore, over time, long-term high-dose therapy may be associated with the development of some adverse effects. Thus, additional thermal spa complementary therapeutic tool mainly for subjects whose symptoms are not adequately controlled with the usual drug-based therapeutic approach seems to be a good option. With supplementary STWs treatment it may be possible to regain symptom control and eventually reduce baseline drug therapy. Another aspect which must be borne in mind is that with STWs treatment at Spas, additional psychological components may also contribute toward clinical improvement. These components include leisure time, opportunity for relaxation, being aware of regular clinical monitoring, and various cultural aspects, all of which may play a part in Spa treatment-associated final results (164).

## Conclusions and Future Perspectives

Mainly due the presence of H_2_S, STWs might be an advantageous and promising option as an add-on non-pharmacological complementary therapy for respiratory diseases, such as AR, chronic rhinitis/rhinosinusitis, bronchial asthma and COPD, since these natural mineral waters have been associated with significant, quick onset, and relatively long-lasting improvement of clinical parameters in patients with these diseases. Furthermore, H_2_S-rich STWs have various effects upon inflammatory and immunological parameters that may contribute toward their clinical efficacy. It may thus be possible to use STWs in a preventive way in terms of disease progression and exacerbations, although this needs to be better ascertained. Moreover, if correctly applied, no significant side effects have been reported with STWs. Nevertheless, in spite of the potential benefits of STWs in respiratory diseases, further elucidation of the mechanisms underlying such benefits warrants further studies. To that end, *in vitro* models appear to be a promising choice for studying the effects of STWs on human respiratory mucosa. Recently, Epithelix Sàrl (Switzerland) and MatTek Corporation (USA) have developed an *in vitro* 3D human lung models with a morphologically and functionally differentiated structure as well as an ability to be maintained in a homeostatic state for a long period of time and to be a ready-to-use model (165, 166). In addition, this 3D cell model can also be used to perform safety testing of occupational and environmental chemicals, pharmaceutical development, drug delivery, and inflammatory responses (167–172). Overall, since these *in vitro* 3D models seem to reproduce human biological responses, they are an interesting choice in the study of various respiratory diseases, such as AR, asthma, and COPD, namely in terms of their response to STWs exposure, and this is one of the current interests of our research, which is in accord with current ethical concerns regarding the use of animals in research investigations, i.e., the 3R (Replace, Reduce, Refine) and animal welfare.

## Contribution of this Review to the Field

This review is novel andignificantly contributes toward having a clearer and more organized view of the role of sulfurous thermal/natural mineral waters (STWs), in inhalational treatment of respiratory diseases, such as rhinitis, asthma or chronic obstructive pulmonary disease. Furthermore, this review also recapitulates the main effects of STWs on lung epithelial-immune crosstalk through the action of its main component, H2S, thereby establishing a relationship between clinical efficacy of these treatments and the underlying immunopathological and immunotherapeutic mechanisms.

## Author Contributions

JV and AE carried out literature searches and wrote the manuscript. EC checked the biochemical aspects. FA checked the immunological aspects. MV and LT-B checked the clinical aspects. All authors made contributions to the review. EC, FA, MV, and LT-B checked the final version of the manuscript.

### Conflict of Interest Statement

The authors declare that the research was conducted in the absence of any commercial or financial relationships that could be construed as a potential conflict of interest.

## Abbreviations

STWs: Sulfurous thermal waters
H_2_S: Hydrogen sulfide
IL: Interleukin
COPD: Chronic obstructive pulmonary disease
MAPK: Mitogen-activated protein kinase
NF-κb: Nuclear factor-kappa B
AR: Allergic rhinitis.
